# Editorial: Impacts of physical activity and exercise on metabolic disorders

**DOI:** 10.3389/fendo.2026.2024969

**Published:** 2026-10-21

**Authors:** Duarte Henriques-Neto, Miguel Peralta, Adilson Marques

**Affiliations:** 1 Research Centre in Sports Sciences, Health Sciences and Human Development (CIDESD), University of Maia, Maia, Portugal; 2 Interdisciplinary Center for Human Performance, Faculty of Human Kinetics, University of Lisbon, Dafundo, Portugal; 3 Institute of Environmental Health, Faculty of Medicine, University of Lisbon, Lisbon, Portugal

**Keywords:** adults, dose-response, exercise, metabolic disorders, physical activity

## Introduction

Non-communicable diseases (NCDs) currently represent the greatest and most complex public health challenge worldwide. According to the World Health Organization, NCDs are responsible for 75% of deaths globally, corresponding to 43 million annual deaths, of which 18 million are considered premature deaths, occurring before the age of 70 (1, 2).

The development of NCDs, such as type 2 diabetes mellitus (T2DM), obesity, arterial hypertension, and polycystic ovary syndrome (PCOS), is intrinsically linked to societal lifestyle factors, including dietary habits, social habits, and the regular practice of physical activity (3–5). Physical inactivity, which currently affects over 36% of adults worldwide, is a major risk factor for the development of these pathologies.

Physical activity and structured exercise have established themselves as fundamental non-pharmacological interventions (6). However, substantial knowledge gaps remain regarding the optimal dose-response relationship, the comparative efficacy of different exercise modalities, the impact of fractionated versus continuous regimens, the psychosocial mediators of adherence, and the integration of digital health platforms.

This special issue aimed to gather multidisciplinary contributions integrating human systems biology and exercise physiology in populations with metabolic disorders, providing a holistic, translational perspective on the role of exercise and digital technologies in cardiometabolic and endocrine health.

## Systemic mechanisms

Exercise modulates gut microbiota, reinforces the enteric barrier, and synchronises circadian metabolic networks to optimise myocardial energy metabolism and mitigate oxidative stress. Pastor-Cisneros et al. confirmed the robustness of these mechanisms at the population level in the Mexico City Prospective Study, following 155,156 adults for over 18 years. The study demonstrated that the practice of physical exercise was consistently associated with a significant reduction in all-cause mortality (12% to 16%), premature mortality under 75 years of age (16% to 20%), and cardiovascular mortality (10% to 15%), maintaining its protective effect even in the established presence of classic risk factors such as hypertension, diabetes, obesity, or smoking.

## Optimisation of modalities, dose-response, and temporal fractionation

For glycaemic control in ageing populations with T2DM, precisely determining exercise volume and type is essential for clinical practice. A meta-analysis evaluated mind-body modalities in T2DM, revealing outcome-specific advantages: Pilates stood out for balance and walking capacity; walking meditation excelled for aerobic fitness; yoga for muscle strength gains; and mindfulness interventions for reducing anxiety (Zhan et al.).

Regarding the temporal distribution of exercise, Zhang et al. compared accumulated exercise (fractionated into short bouts throughout the day, such as ‘exercise snacks’) with traditional continuous exercise of a corresponding volume. Using equivalence testing (TOST), they showed equivalence between the two approaches for chronic outcomes such as body fat percentage, lean mass, waist circumference, and plasma lipids, validating fractionation as a viable alternative for individuals with severe time constraints.

In young university adults, Sun et al. compared high-intensity interval training (HIIT) with moderate-intensity continuous training (MICT) and found significantly greater reductions in body weight, fat percentage, waist circumference, and fat mass with HIIT, highlighting its time efficiency in managing adiposity.

## Psychosocial determinants, reproductive health, and digital interventions

The sustainability of lifestyle changes depends on cognitive and behavioural processes. Su et al. examined adults and older adults with T2DM and found, through structural equation modelling, that physical activity indirectly influences quality of life via exercise self-efficacy and the sequential pathway between psychological resilience and self-efficacy, showing that psychological empowerment is indispensable for maintaining active behaviour.

In the endocrine and reproductive spectrum of PCOS, multimodal interventions are assuming increasing relevance. The systematic review and meta-analysis by Zeng et al. (2026) found that digital health solutions (mobile applications, online platforms, and remote monitoring) improve body mass index, waist circumference, and health behaviours while reducing anxiety and depression in women with PCOS. Similarly, Gautam et al. synthesised the therapeutic value of yoga and mindfulness, highlighting benefits in regulating the hypothalamic-pituitary-ovarian axis, improving insulin sensitivity, reducing circulating androgens, and attenuating psychological stress in this pathology.

## Conclusion

The evidence in this special issue supports physical activity, adapted exercise, and digital interventions as essential therapeutic tools for preventing and controlling cardiometabolic and endocrine diseases. Integrating these approaches into healthcare systems’ daily routines, through individualised prescriptions that respect dose-response thresholds, individual preferences, and behavioural support, is vital to mitigate premature mortality and reduce global economic healthcare burdens.
